# A Machine Learning Tool to Predict Survival After First Surgery in Peripheral Artery Disease Patients

**DOI:** 10.1007/s12265-025-10692-1

**Published:** 2025-10-08

**Authors:** Martina Doneda, Ettore Lanzarone, Fabio Riccardo Pisa, Bianca Pane, Giovanni Pratesi, Giovanni Spinella

**Affiliations:** 1 Department of Electronics Information and Bioengineering (DEIB), Politecnico Di Milano, Milan, Italy; 2 Department of Management, Information and Production Engineering (DIGIP), University of Bergamo, Dalmine (Bg), Italy; 3 Institute for Applied Mathematics and Information Technologies (IMATI), National Research Council of Italy (CNR), Milan, Italy; 4 Department of Surgical and Integrated Diagnostic Sciences (DISC), University of Genoa, Viale Benedetto XV 6, Genoa, Italy; 5 Vascular and Endovascular Surgery Clinic IRCCS Ospedale Policlinico San Martino, Genoa, Italy

**Keywords:** Peripheral artery disease, Mortality, Prediction, Machine learning

## Abstract

The aim of this study was to develop and validate a machine learning tool for predicting survival in PAD patients who received surgical treatment. We used the data from 1,615 patients who underwent PAD surgery from 2005 to 2020. Gradient boosted decision trees (GBDTs) were used to predict mortality at one, three and five years after the first surgery, while predictor importance was assessed using the SHAP values method. The area under the curve (AUC) of the receiver operating characteristic curve of the one-, three and five-year prediction models were 0.86, 0.84 and 0.80, respectively. Disease stage was the most important predictor, along with age, chronic kidney disease status, hospital length-of-stay and total number of comorbidities. Presence of dyslipidemia was slightly predictive of one- and three-year mortality. Simple clinical and demographic parameters can be used to train a GBDT model capable of predicting PAD follow-up mortality.

**Graphical Abstract:**

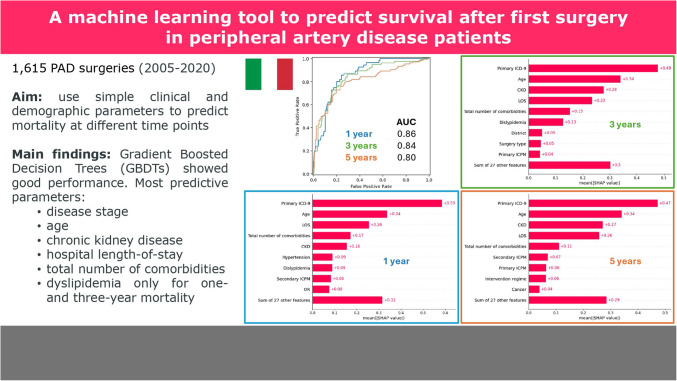

## Introduction

Peripheral arterial disease (PAD) is a widespread and debilitating condition characterised by heterogeneous presentations and symptoms that affects quality of life and limb survival and can even lead to patient death [1, 2]. Although the clinical stage and type of treatment for symptomatic patients may be very different, the presence of PAD alone is a risk factor for long-term survival [3]. In PAD patients with chronic limb-threatening ischemia (CLTI), life expectancy is one factor to consider when evaluating treatment options [4]. Even with less severe clinical presentations, such as intermittent claudication (IC), PAD can result in limb loss and impact patient survival [5, 6]. Therefore, in addition to assessing the impact of cardiovascular risk factors, it is important to predict the survival of patients with PAD [7, 8].

Several mathematical tools are available to analyse patients with this condition [9–12], and current guidelines for CLTI patients suggest the use of different specific tools [4]. Some focus on validating hypotheses, without taking into account the possibility of making predictions [13]. Other tools leverage machine learning (ML) algorithms to make predictions about patients’ future conditions and survival. Callegari et al. [14] recently reported excellent predictions using an ML tool trained on data from the large Medicare-linked Vascular Quality Initiative (VQI) registry. Gupta et al. [15] also developed a predictive model with good accuracy, leveraging a sample of almost 10,000 patients.

AI and ML are transforming medicine by outperforming traditional methods in predicting clinical outcomes [16], such as risk scores or guideline criteria [17]. However, these powerful technologies face a significant hurdle in the form of their need for large amounts of data to be trained effectively, such as registries from several facilities with homogeneous characteristics. This issue, coupled with models’poor accuracy when applied to patient populations different from those they were trained on, has limited their widespread adoption. Data interoperability between different health systems and hospitals is a major issue and is not easy to achieve, which prevents the creation of predictive ML tools from large patient populations. Therefore, it would be advisable to develop and validate ML tools trained with smaller populations, to enable their effective use even in facilities that have their own historical data but in limited numbers.

In light of this, the goal of this study was to develop and validate an ML tool for predicting survival in surgically treated PAD patients, using a population from a single centre (therefore limited in size) as training.

We used data from a dataset that was limited in terms of the number of patients evaluated compared with larger datasets from multicentre trials [14]. At the same time, the data refer to an older population, reflecting the massive ageing of the European population, consistent with future scenarios [18].

As explainability of ML models is increasingly a key requirement for tracking predictions and improving end-user (physician) confidence and adoption in clinical practice, we evaluated the importance of the different predictors included in the proposed ML models. Finally, we considered a set of Kaplan-Meier estimator curves with stratifications for a subset of predictors, and compared them using a multivariate log-rank test to assess whether the curves for different values of a given predictor were statistically significant.

## Methods

We conducted a retrospective analysis of all patients who underwent PAD surgery from 2005 to 2020 at the Vascular and Endovascular Surgery Clinic of the IRCCS Ospedale Policlinico San Martino, Genoa, Italy. Data were processed in compliance with the Declaration of Helsinki.

### Dataset

Data were extracted from the database of the internal management software of the analysed surgery clinic. Interventions reported in the database were aggregated by patient. The following inclusion and exclusion criteria were applied:

- All patients who had their first lower-limb surgery for PAD between 2005 and 2020 were considered in the study.
- Patients were stratified based on principal discharge diagnosis by ICD-9 codes [19], and only patients with codes of 440.21 (*atherosclerosis of native arteries of the extremities with intermittent claudication*), 440.22 (*atherosclerosis of native arteries of the extremities with rest pain*), 440.23 (*atherosclerosis of native arteries of the extremities with ulceration*) and 440.24 (*atherosclerosis of native arteries of the extremities with gangrene*) were included.
- Patients with erroneous or missing data were excluded.

A total of 1,615 patients were included in the study. The distribution of their first surgery was almost uniform over the years: 78 in 2005 (4.83%); 124 in 2006 (7.68%); 69 in 2007 (4.27%); 78 in 2008 (4.83%); 57 in 2009 (3.53%); 71 in 2010 (4.40%); 90 in 2011 (5.57%); 90 in 2012 (5.57%); 95 in 2013 (5.88%); 105 in 2014 (6.50%); 104 in 2015 (6.44%); 94 in 2016 (5.82%); 103 in 2017 (6.38%); 123 in 2018 (7.62%); 114 in 2019 (7.06%); 117 in 2020 (7.24%) and 103 in 2021 (6.38%).

This dataset is the same one considered in a previous descriptive analysis [20], and further details on the collected data can be found there.

Data on age, sex, PAD clinical stage and known risk factors were collected for each patient at the date of the first intervention. Risk factors included coronary artery disease (CAD), carotid artery stenosis (CAS), hypertension, chronic obstructive pulmonary disease (COPD), diabetes, dyslipidemia, chronic kidney disease (CKD), presence of aortic aneurysm, history of stroke and presence of cancer. These were derived from diagnosis codes.

For the surgery itself, data on anatomical location, type and anaesthesia type (general, spinal or local) were collected. Anatomic location was classified into aorto-iliac, femoral, above-knee popliteal, below-knee popliteal, foot, leg vessels and multilevel. Treatment type was classified as open, endovascular, hybrid or primary amputation.

For the procedures performed in the first surgery, International Classification of Procedures in Medicine (ICPM) codes were used as a proxy of surgery complexity [21]. There were 35 unique primary ICPM codes; 638 records also presented also a secondary ICPM code (32 unique), 390 a tertiary code (24 unique), 187 a quaternary code (21 unique), 104 a quinary code (16 unique) and 59 a senary code (9 unique). The same ICPM codes could be assigned in different positions in different patients; these data were retained because they are indicative of patients’ surgical complexity.

Hospital length of stay (LOS) and administrative data for first surgery were also collected, including destination (recovery room, ward, resus, ICU, high-dependency unit).

Summary statistics for the analysed population are shown in Table 1. Numerical variables are reported as median and interquartile range because none were normally distributed (*p*-values of the Shapiro-Wilk test < 0*.*05). Categorical variables are reported as numbers and percentages, and binary variables are reported with the prevalence of Boolean value “True”. All listed variables were included in the ML models as predictors.

**Table 1:** Predictors used in the ML model and summary statistics for the patients included in the study

**Population characterization**	
**Demographic data**	**Summary**
Sex	Males: 1089 (67.43%), Females: 526 (32.57%)
Age in years	74 (60–80)
**Comorbidities**	**Summary**
Aneurysm	64 (3.96%)
Stroke	32 (1.98%)
CAS	358 (22.17%)
CAD	338 (20.93%)
Hypertension	924 (57.21%)
COPD	63 (3.90%)
Diabetes	511 (31.64%)
Dyslipidemia	299 (18.51%)
CKD	228 (14.12%)
Cancer	98 (6.07%)
n° total comorbidities	4 (3–5)
**Surgical data**	**Summary**
LOS	9 (5–20)
Surgery type	Open: 816 (50.53%); Endovascular: 431 (26.69%); Hybrid: 128 (7.93%); Amputation: 234 (14.49%); Revision: 6 (0.37%)
Operated district	Aortoiliac: 425 (26.32%); Femoral: 209 (12.94%); Above-knee popliteal: 320 (19.81%); Below-knee popliteal: 107 (6.63%); Leg vessels: 56 (3.47%); Foot: 131 (8.11%); Multilevel: 367 (22.72%)
Side	Left: 684 (42.35%); Right: 685 (42.41%); Bilateral: 246 (15.23%)
Anesthesia	General: 863 (53.44%); Spinal: 516 (31.95%); Local: 236 (14.61%)
**Administrative data**	**Summary**
Intervention regime	Elective: 1,544 (95.60%), Emergency: 71 (4.40%)
Destination after surgery	Recovery room: 876 (54.24%), Ward: 702 (43.47%), ICU 36 (2,23%)
Primary ICD-9 code	440.21: 699 (43.28%), 440.22: 239 (14.80%), 440.23: 151 (9.35%), 440.24: 526 (32.57%)
Presence of secondary ICPM	39 (1.18%)
Presence of secondary ICPM	638 (39.50%)
Presence of tertiary ICPM	390 (24.15%)
Presence of quaternary ICPM	187 (11.58%)
Presence of quinary ICPM	104 (6.44%)
Presence of senary ICPM	59 (3.65%)
n° total ICPM codes	1 (1-2)

The predictive performance of the proposed ML models for mortality were assessed at one, three and five years after the first surgery. All-cause mortality was assessed for each patient at these three follow-up time points from the first surgery, using hospital electronic medical records. A mortality variable was introduced for each patient and follow-up time point, equal to 1 if the patient died within that time period, or 0 if the patient survived at least to that point. Patients were excluded from the analysis at a given time point if the interval between their first surgery and the data collection date was shorter than the designated follow-up period. For example, patients observed for 3.5 years were excluded from the analysis at five years but included for the analyses at one and three years. This way, we were able to track 253 patients who died in the first year out of a total of 1,484 observed (17.0%), 411 who died within the third year out of 1,299 (31.6%), and 523 who died within the fifth year out of 1,152 (45.4%).

A stratified version of Table 1, showing data stratified for surviving and deceased patients at each considered follow-up time point, is provided in the Supplementary Materials of this article.

### ML Models

The data included several categorical predictors. Therefore, we applied gradient boosted decision trees (GBDTs), a supervised ML technique that has proven to be very effective in such situations [22]. GBDT is an ensemble ML technique that can be used both for regression and classification tasks. It evaluates the predictive performance of multiple sequential decision trees to improve model accuracy.

A cross-validation scheme was applied for each follow-up period, splitting the dataset in an 80-20 fashion, with 20% of the data separated to be used as test data to externally validate the fitted model. Internal cross-validation was also applied during model training with a *k*-fold approach with *k* = 3 over 1,000 repetitions with random split and shuffle.

Model predictive performance at each follow-up period was evaluated in terms of the confusion matrix, from which accuracy and other relevant metrics were derived, including sensitivity or true positive ratio (TPR), specificity or true negative ratio (TNR), balanced accuracy, positive and negative predicted values (PPV and NPV), false positive and negative ratios (FPR and FNR) and F1-score. Moreover, predictive performance was evaluated in terms of the area under the curve (AUC) of the receiver operating characteristic (ROC) curve.

Moreover, we utilized Shapley additive explanation (SHAP) values to analyse the influence of each predictor on the outcome of the model [23]. In the context of explainability, SHAP values are used to assign each predictor an importance value with respect to the prediction task. They help to understand the influence of each predictor on model outcome [24], which is important for practitioners. The model and general data management architecture were developed in Python, using open-source libraries.

### Kaplan-Meier Curves

To further investigate the relevance of important predictors, as identified by their SHAP value, we produced a set of Kaplan-Meier curves and compared with each other using a multivariate log-rank test. The goal was to assess whether the curves for different values of a given predictor were statistically significant. The curves and tests were again performed in Python, using open-source libraries.

## Results

### ML Models

**Fig. 1:**
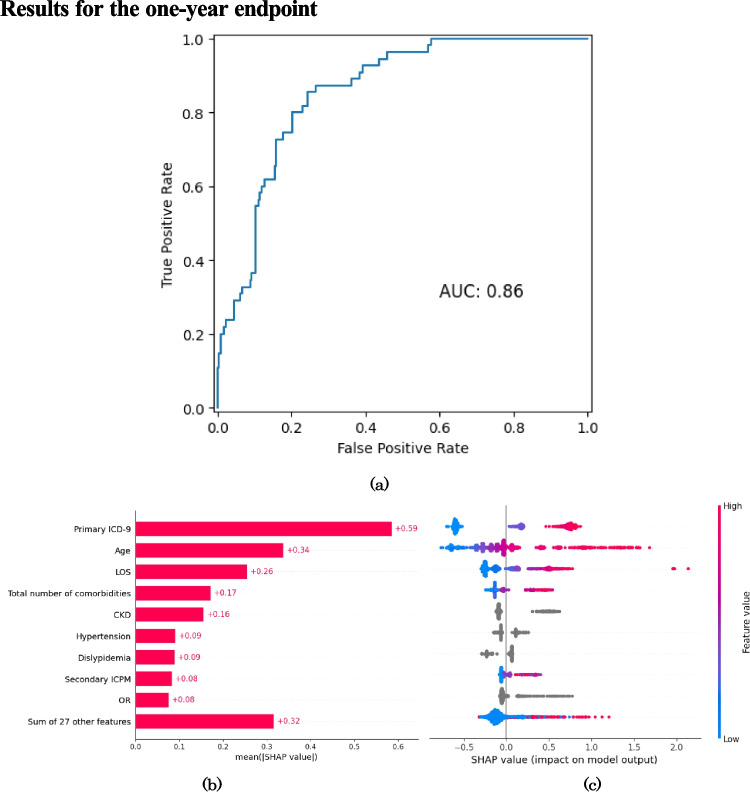
ROC curve (**a**), SHAP values (**b**) and SHAP beeswarm plot (**c**) for one-year all-cause mortality

**Fig. 2:**
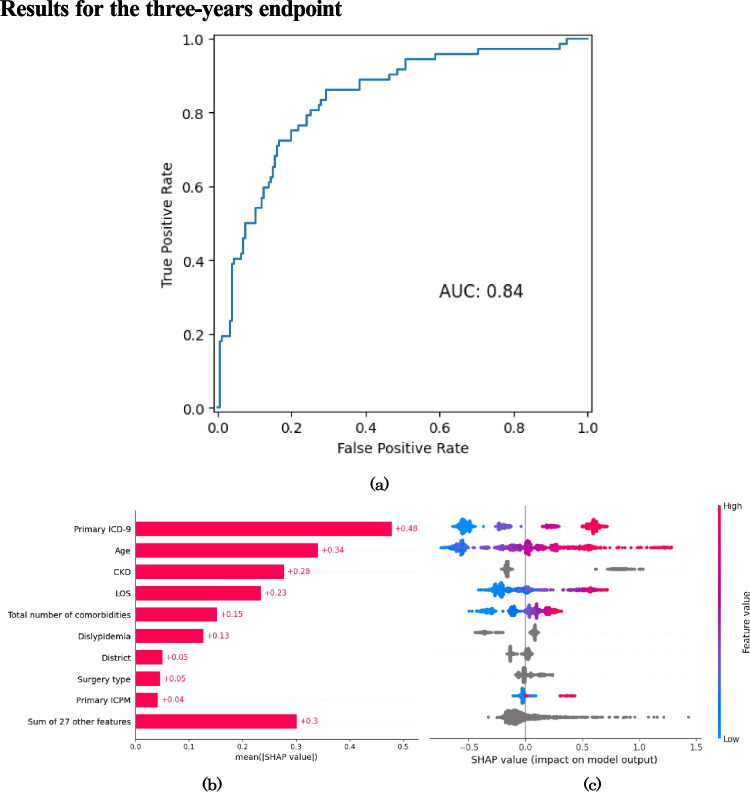
ROC curve (**a**), SHAP values (**b**) and SHAP beeswarm plot (**c**) for three-year all-cause mortality

**Fig. 3:**
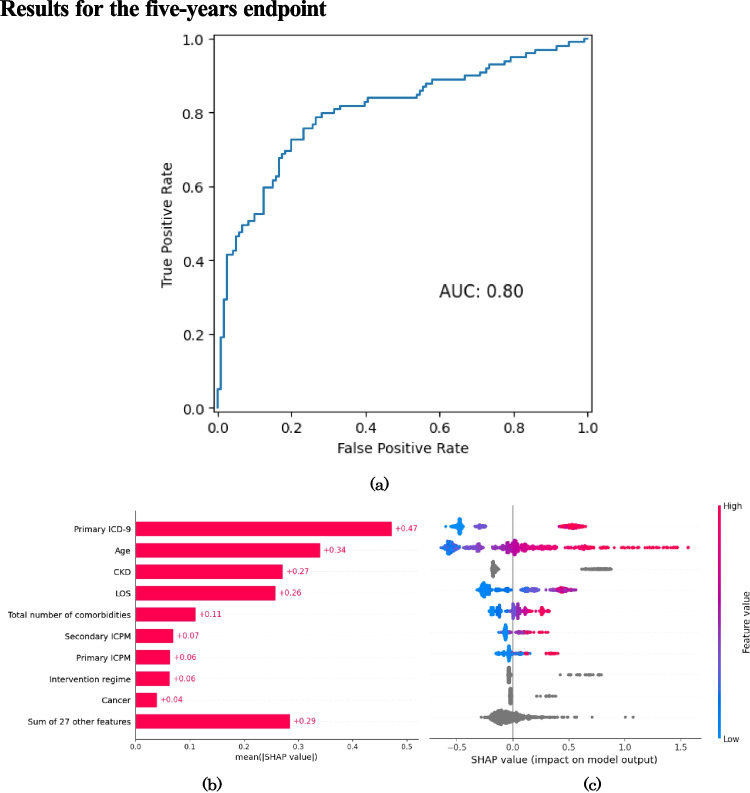
ROC curve (**a**), SHAP values (**b**) and SHAP beeswarm plot (**c**) for five-year all-cause mortality

The performance metrics of the GBDT models at each follow-up time point are presented in Table 2, which reports accuracy, sensitivity, specificity, balanced accuracy, PPV, NPV, FPR, FNR, F1-Score and AUC. Moreover, the corresponding confusion matrices on the test (holdout) set are reported in Table 3. The ROC curves for the ML models fit at each endpoint are reported in Figures 1(a), 2(a) and 3(a). Alongside the ROC curves, each figure also contains a SHAP bar plot (b) and a SHAP bee-swarm plot (c) to visualise the relative importance of predictors.

### Kaplan-Meier Curves

**Fig. 4:**
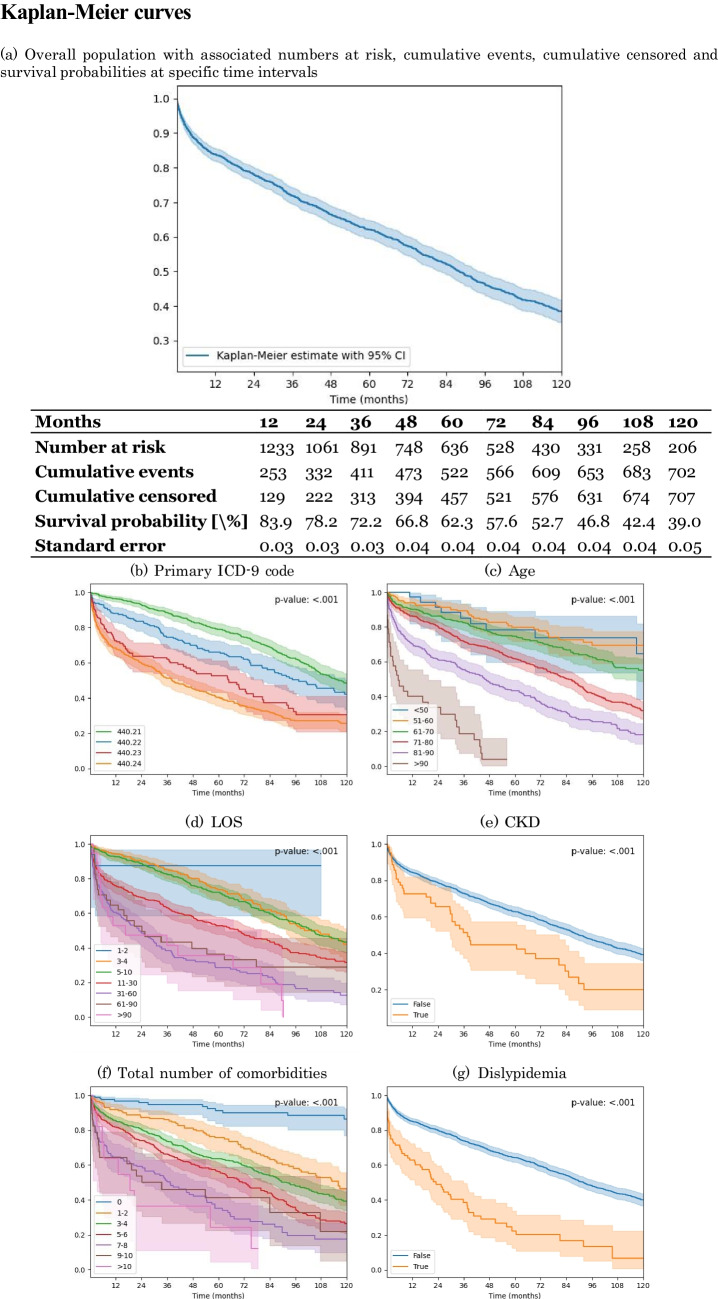
Kaplan–Meier survival curve of the overall considered population (**a**), and Kaplan–Meier survival curves stratified by predictors with a SHAP value ≥ 0.10 in any of the three fitted models, that is: (**b**) according to the primary ICD-9 code assigned to each patient, (**c**) their age group, (**d**) their total LOS, binned, (**e**) their CKD status, (**f**) their total number of comorbidities and, finally, (**g**) their dislypidemia status

A Kaplan-Meier curve showing the overall in the considered patient population is reported in Figure 4. Note that the values reported for number at risk at months 12, 36 and 60 (corresponding to one, three and five years) differ slightly from the number of patients used in the ML models. This is due to the fact that Kaplan-Meier estimators do not use the same methodology we employed to determine inclusion in the analysis at the different time points, as explained at the end of Section 2.1. Figure 4 also reports the Kaplan-Meier curves for a series of stratifications. The predictors for which to stratify for were chosen among the ones that had a SHAP value of at least 0.10 in any of the predictive models fitted for the considered endpoints. For the three predictors with an integer domain (i.e. total number of comorbidities, age and LOS), values were binned in order to enhance the visual interpretability of the plots. The shaded area in each curve represents the 95% confidence interval of the Kaplan-Meier estimator, and the *p*-value of the multivariate log-rank test comparing the groups is also reported in the plot.

**Table 2:** ML model performance for the three follow-up periods. TP, TN, FP and FN denote the true positive, true negative, false positive and false negative predictions in the test set, respectively

Metric	Formula	Follow-up period		
		One year	Three years	Five years
Accuracy	$\frac{TP + TN}{TP + FP + TN + FN}$	81.56%	78.54%	75.45%
Sensitivity (TPR)	$\frac{TP}{TP + FN}$	23.64%	54.17%	72.73%
Specificity (TNR)	$\frac{TN}{FP + TN}$	95.59%	88.57%	77.69%
Balanced accuracy	$\frac{TPR + TNR}{2}$	59.62%	71.37%	75.21%
Precision (PPV)	$\frac{TP}{TP + FP}$	56.52%	66.10%	72.73%
NPV	$\frac{TN}{TN + FN}$	83.78%	82.45%	77.69%
FPR	$\frac{FP}{FP + TN}$	4.41%	11.43%	22.31%
FNR	$\frac{FN}{FN + TP}$	76.36%	45.83%	27.27%
F1-Score	$\frac{2TP}{2TP + FP + FN}$	33.33%	59.54%	72.73%
AUC	$\text{integral from the figure}$	0.86	0.84	0.80

**Table 3:** Confusion matrices obtained on the test set for the threee models

		Predicted					
		Alive	Dead	Alive	Dead	Alive	Dead
Observed	Alive	217	10	155	20	94	27
	Dead	42	13	33	39	27	72
		One year		Three years		Five years

## Discussion

A predictive model for the survival of patients with PAD after treatment was developed using ML. The use of ML and artificial intelligence in healthcare has been rising quasi-exponentially over the years.

Habehh and Gohel [25] recently published a review that contains a useful, clinician-oriented overview of the different types of approaches that can be used in various relevant medical fields. They also explored the importance of making a model explainable, as it aids decision-makers in making reasonable, data-driven decisions for personalised patient care.

Using ML to predict mortality has already been suggested in the literature. Several studies have evaluated the risk of death and amputation in patients with PAD after revascularisation, and one of the main characteristics of ML models is related to the large number of patients that need to be included to obtain satisfactory results. As reported by Liu et al. [26], Gupta et al. [15] had one of the largest number of cases in the field to date, with 9,556 PAD patients who underwent infrainguinal bypass surgery included. They identified seven predictors associated with 30-day mortality using standard logistic regression analysis: increasing age, systemic inflammatory response syndrome, chronic corticosteroid use, chronic obstructive pulmonary disease, dependent functional status, dialysis and lower extremity rest pain. The predictive model showed a C-index equal to 0.81. Other studies in the literature have demonstrated the effective development of models with good predictive performance even with small samples; for example, Kobayashi et al. [27], Azuma et al. [28] and Roijers et al. [29] included 185, 520 and 449 patients respectively. Kobayashi et al. [27] established a predictive scoring system for one-year amputation-free survival and ulcer healing rate with an AUC of 0.92 in patients treated with endovascular surgery. Infected wound, dependence on hemodialysis, no visible blood flow to the wound, and major tissue loss were found to be independent risk factors. Azuma et al. [28] used multivariate Cox regression analysis to identify old age, impaired mobility, low body mass index, renal failure, heart failure, and high WIfI grade [30] as independent risk factors for all-cause mortality (all with *p*-value< 0*.*05). Their area under the time-dependent receiver operating characteristics curve was equal to 0.83 for thirty-day mortality and 0.81 for two-year mortality. Roijers et al. [29] built a predictive model for the six-month mortality of elderly patients undergoing surgical or endovascular treatment, and showed that age, living in a nursing home, physical impairment, and high ASA class had the strongest association with increased mortality. Their area under the receiver operating characteristic curve of the six-month mortality model was equal to 0.81 (*p*-value < *.*001). In these studies, although the predictive performance was comparable with that on larger samples, the applicability of the system remains to be further validated given the limited patients. Furthermore, in all the predictive models mentioned, patients are often referred to the CLTI group only.

One of the most recent studies is by Callegari et al. [14], in which they made mortality predictions after surgical intervention over a cohort of 10,114 patients, achieving an accuracy of 70.03% for the three-year time point. To make a further comparison with the existing literature, Ross et al.[31] created a random forest model for the prediction of long-term mortality, using a database of a similarly-sized cohort to ours, consisting of 1,755 patients enrolled via the Genetic Determinants of Peripheral Arterial Disease (GenePAD) study, performed in the United States. They did not report mortality prediction at a specific time point, but generally referred to *future* mortality by the end of their study, which lasted eight years. Median follow- up time for the included patients was 5.3 years, and they obtained an AUC for mortality prediction equal to 0.87.

In our study, the achieved values of sensitivity and specificity were 23.64% and 95.59%; 54.17% and 88.57%, and 72.73% and 77.69% for the one-, three- and five-year prediction models, respectively. Also, the AUC of the one-, three- and five-year prediction models were equal to 0.86, 0.84 and 0.80, respectively. Comparison with the cited articles is summarized in Table 4.

The innovative aspect is that we achieved these results using only data from a single hospital centre. Although the number of patients is small compared to the samples of the cited works, their treatment in a single centre considerably reduced the risk of bias related to the potentially different performances of the operators, the difference in availability of materials, and the different peri-operative management protocols of different centres. Furthermore, the predictive performance of our model outperformed that of Callegari et al. [14], especially at the three- year time point, with roughly one sixth of the patients. In addition, our predictive model was built to predict mortality up to five years, differently from the cited works in which the time points were calculated on shorter time periods.

This approach demonstrates that highly effective predictive models can be created even with limited patient populations, overcoming the big data barrier and making AI accessible and applicable in local contexts. This will allow us to pave the way for a new generation of personalised, precise ML tools that can be successfully implemented in healthcare facilities with limited historical data, thus democratising the use of these life-saving technologies.

In articles analysing mortality or survival during follow-up, this outcome is divided according to clinical stage (CI and CLTI). Mortality during follow-up is always higher in patients with CLTI. Levin et al. [32] performed a retrospective cohort analysis, querying the VQI registry from January 1, 2010 to May 31, 2021 for peripheral vascular intervention (PVI), infrainguinal bypasses (IIB), and suprainguinal bypasses (SIB) for IC and CLTI across 286 US centres. They included 31,457 PVIs (44.7% IC, 55.3% CLTI), 7,978 IIBs (26.9% IC, 73.1% CLTI) and 2,149 SIBs (50.1% IC, 49.9% CLTI) recorded in the VQI. Five-year mortality after PVI was 37.2% and 71.1% for IC and CLTI, respectively; after IIB it was 37.8% and 60% for IC and CLTI, respectively; and after SIB it was 33.8% and 53.8% for IC and CLTI, respectively. For this reason, we decided to take into account the clinical stage of the disease as one of the factors that may affect mortality during follow-up. Our results confirmed that, in patients treated for PAD, the most severe clinical stage always has a significant impact on one-, three- and five-year mortality. Other factors had a different effect on the mortality rate during the follow-up period. In fact, the presence of dyslipidemia was a significant risk factor for one- and three-year mortality, while cancer was only marginally significant for five-year mortality. Hypertension was only slightly significant for one-year mortality. However, it was the total number of comorbidities that had the most importance for all considered time points, as also clearly highlighted in Figure 4(f).

Comorbidities are very important in determining the clinical progression of PAD patients. Rantner et al. [33] prospectively followed 255 male patients with intermittent claudication from the CAVASIC Study for seven years for overall mortality, vascular morbidity and mortality, and local PAD outcomes. Overall mortality was 16.1% (*n*=41); most patients died from cancer (*n*=20), and half of the dead patients (*n*=22; 8.6%) died within the first five years. We observed an aneurysm incidence of 3.96% and a neoplasia incidence of 6.07% in the study population, which is similar to a recent meta-analysis by Villemur et al. [34]. This may be due to smoking, which is a common risk factor for both cancer and PAD [35].

When the type of revascularisation was taken into account, the highest survival rates were observed in patients who underwent open surgery. It is likely that the patients who were considered fit for open surgery had better performance status and that the observed survival was a consequence of this. It is interesting to note that long-term survival was higher in patients who underwent direct amputation (without revascularisation) than in the group of patients who underwent endovascular revascularisation. This needs to be considered given the long period of the study. In the past, endovascular treatment was considered a treatment for high-risk patients [36]. The lower survival during follow-up probably reflects this. The presence of various cardiovascular risk factors such as hypertension, heart disease, diabetes and chronic kidney disease correlates with poorer survival over the entire follow-up period. However, the predictive model for mortality at one, three and five years showed that the impact of a single risk factor can differ according to the duration of follow-up.

The main limitation of this study is that we collected data retrospectively over a long period of observation, and this must be taken into account because therapeutic strategies, especially endovascular approaches, are constantly evolving and an improvement in outcome in terms of limb salvage is certainly expected. For this reason, we decided to only evaluate mortality during follow-up. Another limitation concerns the retrospective nature of the study, in which inclusion and exclusion criteria were defined a posteriori when patients’ data had already been collected.

**Table 4:** Comparison with the literature

Author	N° of patients	Predictive period	AUC at 30 days	AUC at 6 months	AUC at 1 year	AUC at 2 years	AUC at 3 years	AUC at 5 years
[15]	9,556	30 days	0.81	/	/	/	/	/
[27]	185	1 year	/	/	0.92	/	/	/
[31]	1,755	5 years	/	/	/	/	/	0.87
[28]	520	30 days, 2 years	0.83	/	/	0.81	/	/
[29]	449	6 months	/	0.81	/	/	/	/
[14]	10,114	3 years	/	/	/	/	0.70	/
*Our work*	*1,615*	*1, 3 and 5 years*	*/*	*/*	*0.86*	*/*	*0.84*	*0.80*

## Conclusions

Simple clinical and demographic parameters can be used to train a ML model capable of predicting PAD mortality at one, three and five years with satisfactory performance, comparable to ML models built with larger samples. Disease stage is the most important predictor, along with age, hospital length of stay and total number of comorbidities. The predictive capability of the model can be useful in proposing the best treatment for the patient based on clinical presentation, comorbidities and life expectancy, as underlined by the latest guidelines of the European Society for Vascular Surgery[6] and applicable to the Italian population.

## Data Availability

All data generated or analysed during this study are included in this published article.

## Abbreviations

AUC: Area under the curve
CAD: Coronary artery disease
CAS: Carotid artery stenosis
CKD: Chronic kidney disease
CLTI: Chronic limb-threatening ischemia
COPD: Chronic obstructive pulmonary disease
FNR: False negative ratio
FPR: False positive ratio
GBDT: Gradient-boosted decision tree
IC: Intermittent claudication
ICD-9: International Classification of Diseases, 9^th^ edition
ICPM: International Classification of Procedures in Medicine
ICU: Intensive care unit
IIB: Infrainguinal bypass
LOS: Length of stay
ML: Machine learning
NPV: Negative predicted value
PAD: Peripheral artery disease
PPV: Positive predicted value
PVI: Peripheral vascular intervention
ROC: Receiver operating characteristic
SHAP: Shapley additive explanation
SIB: Suprainguinal bypass
TNR: True negative ratio
TPR: True positive ratio
VQI: Vascular Quality Initiative
